# Doppler sonography for evaluation of hemodynamic characteristics of fetal umbilicus in Beetal goats

**DOI:** 10.14202/vetworld.2015.412-416

**Published:** 2015-03-28

**Authors:** Kailash Kumar, Ramesh K. Chandolia, Sandeep Kumar, Tarachand Jangir, Ram Avatar Luthra, Sonu Kumari, Sarvan Kumar

**Affiliations:** 1 Department of Veterinary Gynaecology & Obstetrics, Lala Lajpat Rai University of Veterinary and Animal Sciences, Hisar, Haryana, India; 2 Department of Veterinary Pathology, Lala Lajpat Rai University of Veterinary & Animal Sciences, Hisar, Haryana, India

**Keywords:** Doppler sonography, goats, gestation period, umbilical cord

## Abstract

**Aim:**

The objective of this study was to evaluate the hemodynamic characteristics of umbilical vessels in healthy pregnant Beetal goats.

**Materials and Methods:**

Doppler examinations were performed from day 20 to 120 of gestation, twice in week from day 20 to 60 and once in week from day 60 to 120 of gestation on six goats.

**Results:**

Free floating umbilical cord was identified on day 39 of gestation. The umbilical cord waveform was characterized by the simultaneous presence of arterial and venous flow. The pattern of blood flow in the umbilical artery was represented as saw tooth pattern above the baseline. Pattern of blood flow in umbilical vein was flat and wavy in nature; presented below the baseline. Peak systolic velocity (PSV) increased significantly from day 39 to 67 and further between 98 and 120 days of gestation (p<0.05), but there was no significant increase or decrease in end-diastolic velocity (EDV). Pulsatility index (PI) value was increased significantly during 42 to 48 days of gestation and decreased significantly from 98 to 105 days of gestation. On other days, there was no significant increase or decrease. Value of resistance index (RI) was more stable than PI values as there was no significant increase or decrease in RI value.

**Conclusions:**

From the present study, it is reasonable to conclude that the normal blood flow parameters like PI, RI, PSV and EDV during gestation might be helpful in assessment of antenatal development of fetus in the goat. This work provides the basis for further contribution in diagnosing and monitoring high-risk pregnancy in this species.

## Introduction

The goat is an important small ruminant, popularly known as poor man’s cow in India. Previously several studies had used two-dimensional ultrasonography to diagnose pregnancy and for measuring fetal dimension [1,2]. Doppler ultrasound during gestation provides pattern of blood flow in organ of interest such as velocity, circulation and type of blood (arterial or venous) that has important clinical gynecological implications for human and animal species [3].

In domestic animals, Doppler ultrasonography was first used in pregnant sheep as a model for human pregnancy [4]. Recently a few studies using this technique had done in pregnant mares [5], cows [6,7] and bitches [8,9]. Until now, the Doppler technique has not been fully applied in goat-obstetrics and Doppler predictors of abnormal gestation have not been identified. Vascular system in umbilical cord adapts to hemodynamic changes to ensure blood supply to developing placenta and fetus [10]. With this non-invasive technique (Doppler ultrasonography) umbilical artery and venous blood flow pattern were investigated. Only recently, a trans-abdominal approach for Doppler evaluations in anesthetized animals fixed in recumbency has been reported [11]. Experiments were done twice during gestation, after midline laparotomy, surgical mobilization of the uterine arteries and placing a transit-time ultrasonic flow probe around the uterine artery supplying the pregnant horn [11,12]. Color Doppler ultrasonography had been done to characterize blood flow pattern during entire pregnancy in Boer × German improved fawn goat [13]; however, no information is available on blood flow to uterus and developing fetus in tropical breeds. Measurements of the Doppler indices pulsatility index (PI) and resistance index (RI) include calculation of peak systolic velocity (PSV) and the time average maximum velocity (TAMV) values over the time of the cardiac cycle. As gestation advances, in women, PI and RI decrease progressively, indicating an increase in fetal blood perfusion [14]. The velocity of blood flow usually changes according to the Doppler angle, but the A/B ratio (PSV/end diastolic velocity [EDV]) is independent of this angle [15]. A decreasing A/B ratio with advancing pregnancy indicates a decrease of vascular impedance and an increase of vascular perfusion.

Only in some studies in goat fetuses [16] and sheep fetuses [13,17], the umbilical artery has been examined noninvasively in singleton and multiple pregnancies. Moreover, the uteroplacental blood flow is increasing in pregnant ewes between day 46 and day 130 of gestation [17].

The aim of the current study was to assess umbilical artery Doppler parameters like PI, RI, PSV and EDV during gestation that might be used for assessment of antenatal development of the fetus.

## Materials and Methods

### Ethical approval

The study was conducted after the approval of the Institutional Animal Ethics Committee.

### Animals

Six healthy pregnant goats of Beetal breed approx. 2 year of age, weighing approx. 25 kg having history of normal reproductive performance were selected for the study. In all the animals, pregnancy was through natural mating. They were kept on grazing as well stall feeding.

### Ultrasonographic examination

**Figure-1:**
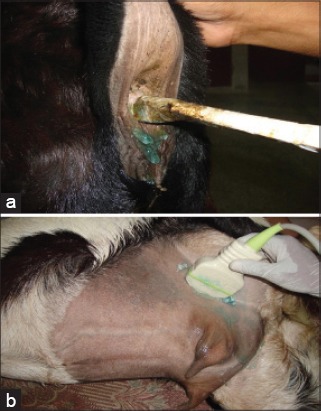
(a) Scanning area for pregnancy monitoring in goats between udder and thigh region, (b) Pre-rectal approach for scanning uterus in early pregnancy. 2D curvilinear trans-rectal probe used for study modified by pvc pipe and adhesive tape for per-rectal approach

Color and pulse Doppler ultrasonography was conducted from day 20 after mating and continued till 4 months of gestation. The ultrasonography was conducted 2 times in a week from day 20 to day 60, then weekly till the end of the experiment. The animals were placed on the operating table by restraining of fore and hind limbs with neck support to lie down on the table on lateral recumbency. No sedation was given to animals. For trans-abdominal ultrasonography of the goat, the scanning area was around teats and lateral abdomen (Figure-1a). The ultrasound machine used for this study was 3D ultrasound machine (Nemio-XG: Toshiba, Japan) having 4D volumetric probe. To the ultrasound machine, computer was attached with software for recording scanning by Doppler ultrasonography.

### Trans-rectal and trans-abdominal probes used

During early pregnancy, 2D intra-operative probe of this machine having frequencies between 5 and 10 MHz was made stiff by fixing it on a 0.5” PVC pipe after slitting it and using adhesive tape (Figure-1b). It was used for trans-rectal ultrasonography and color Doppler study. Later on 2D convex trans-abdominal transducer switchable between 3 MHz to 6 MHz was used for Doppler study (Figure-1a). For the Doppler ultrasonography, 2D probe was selected. Coupling gel was applied on the head of the transducer. Whenever there was a clear image of the conceptus, color Doppler was started. Pulse repetition frequency, color gain, and power were adjusted in order to avoid aliasing. Transducer was moved such that Doppler angle made by the blood flow and direction of the ultrasound beam was minimum. Keeping the transducer constant, pulsed wave Doppler was started and gate size was adjusted such that the sample volume was exactly in the center of blood flow. Identification of umbilical artery and vein was done by color Doppler technique, and further spectral graph was obtained by pulse Doppler technique. Whenever there was clear waveform of the umbilical vessel, color Doppler was switched off leaving the pulsed wave Doppler on the updating two-dimensional image such that the error was minimum. The blood flow of umbilical cord was always recorded. The ultrasound images recorded in the machine were reviewed in the scanner itself to re-examine the images in detail. Waveform in the Doppler ultrasonography of the umbilical vessels was traced manually, and measurements were taken automatically using velocity trace option.

## Results

**Figure 2:**
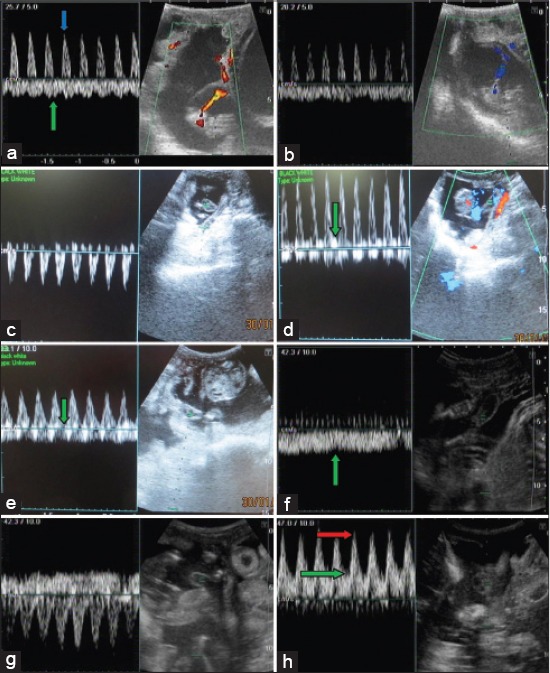
(a) Color Doppler image at day 39 of pregnancy showing blood flow pattern of umbilical artery (blue arrow) and umbilical vein (green arrow) which is present above and below the baseline respectively in spectral mode, (b) Color Doppler image at day 48 of gestation showing blood flow pattern of umbilical artery which is present below the baseline in spectral mode, (c) Color Doppler image at day 60 of gestation showing blood flow pattern of umbilical artery which is present below the baseline in spectral mode. Only systolic wave is present, (d) Color Doppler image at day 67 of gestation showing blood flow pattern of umbilical artery that is present in the baseline in spectral mode. At this day 1^st^ time diastolic component (green arrow) appears along with the systolic wave as shown in image, (e) Pulse Doppler image at day 82 of gestation showing blood flow pattern of umbilical artery and vein which is present above and below the baseline respectively in spectral mode. diastolic component increase in size (green arrow). (f) Pulse Doppler image at day 98 of gestation showing blood flow pattern of umbilical artery and vein that is present above and below the baseline respectively in spectral mode. Pattern of vein is wavy and almost flat (green arrow), (g) Pulse Doppler image at day 112 of gestation showing blood flow pattern of umbilical artery and vein which is present above and below the baseline respectively in spectral mode. Diastolic component umbilical artery increase in size, (h) Pulse Doppler image at day 120 of gestation showing blood flow pattern of umbilical artery and vein which is present above and below the baseline respectively in spectral mode. Diastolic component umbilical artery increase in size and there is no gap between systolic waves of umbilical artery. PSV (red arrow) and EDV (green arrow) are shown in figure.

On 20^th^ day of gestation, both uterine horns showed increased blood supply. The blood coming toward transducer was represented by the pattern above the baseline and vice versa. Free floating umbilical cord was identified on day 39 of gestation. The umbilical cord’s waveform was characterized by the simultaneous presence of arterial and venous flow. The umbilical artery was represented as saw tooth pattern above the baseline with only systolic component and pattern of umbilical vein was flat and wavy in nature, presented below the baseline (Figure-2a). On 48^th^ day of gestation, similar type of blood flow pattern was obtained (Figure-2b). On day 54 of gestation, umbilical artery waveform was almost similar, but both PI and PSV increased significantly (p<0.05). On day 54 and 60 of gestation umbilical artery waveform was identified with only systolic component (Figure-2c). First of all on day 67 of gestation diastolic component of umbilical artery was identified (Figure-2d). On day 76 of gestation, diastolic component of umbilical artery increased further. On day 82 of gestation, both arterial and venous patterns were observed with an increase in diastolic component (Figure-2e). On day 90 of gestation, only venous pattern was seen with continuous flat wavy pattern. Similarly, on day 98 of gestation umbilical vein was focused, and almost similar type of pattern was seen (Figure-2f). On 105^th^ day of gestation, both arterial and venous pattern was seen. In arterial flow, there was more increase in diastolic component. On day 112 of gestation, further increment in diastolic component was observed with similar venous pattern (Figure-2g). On day 120 of gestation, end-diastolic component of artery was increased further, and there was no gap found between wave patterns of artery (Figure-2h). Umbilical vein had wavy margins and typical waveform throughout the study.

Peak systolic velocity increased significantly from day 39 to 67 of gestation from day 98 to 120 days of gestation (p<0.05), but there was no significant increase or decrease in end-diastolic velocity EDV. PI value was increased significantly during 42-48 days of gestation and decreased significantly from day 98 to 105 days of gestation. On other days, there was no significantly increase or decrease. Value of RI has more stable than PI values. There was no significant increase or decrease in RI value.

## Discussion

Nowadays Doppler examinations are used commonly to monitor high-risk pregnancy in human medicine [18-20]. Various fetal blood flow wave form evaluation have been performed to judge the fetal well-being, normal and abnormal placental circulation, intrauterine growth restriction of the fetus and various fetal abnormality during antenatal development. In veterinary medicine there are only few reports that have examined the blood flow pattern in the umbilical cord in goat [16], sheep [17], bitch [8,9,21], and mare [5].

In the present study, umbilicus was detected on day 39 of pregnancy and flow velocity patterns of umbilical vein and artery were recorded. The umbilical artery was the first fetal vessel to be evaluated by Doppler velocimetry in human medicine. In the present study flow velocity waveforms from the umbilical cord have a characteristic saw-tooth appearance of arterial blood flow in one direction and umbilical venous blood flow on the other side of the baseline as continuous wave. Similar findings were observed by Scotti *et al*. [9] in queen. In the current study, the umbilical cord waveforms were identified by the simultaneous presence of arterial and venous flow. This was in agreement with the Scotti *et al*. [9]. The umbilical artery blood flow was characterized only by the systolic waveform until the 67^th^ day of pregnancy, and diastolic component was observed after 67^th^ day of pregnancy. Serin *et al*. [16] detected umbilical artery systolic waveform in goat from day 40 to 85 of gestation, and diastolic wave forms were observed after day 85^th^ of gestation. This difference may be due to breed difference. Scotti *et al*. [9] observed umbilical systolic arterial blood flow at the 5^th^ week before parturition and later on umbilical arterial diastolic waveform was also observed in bitch. The pattern was almost similar to the current study but difference in the days due to the difference in the gestation period between bitch and goat or due to species difference. In our study, color Doppler examination was performed by observing the umbilicus at day 39 of gestation in healthy pregnant Beetal goats. The umbilical artery blood flow was characterized only by the systolic waveform until 69^th^ day of pregnancy. There was not any abnormal blood flow observed during this study such as reverse flow. The directions of arterial blood flow on spectral graph depend upon the position of the transducer. Sometimes a wave below the baseline were the artery and above was the vein in present study because fetus was lying such that flow of artery was away from the transducer, and that of vein was toward transducer. Any flow toward the transducer in pulse Doppler will be depicted above the baseline and away from the transducer below the baseline.

PSV almost increased significantly from day 39 to 120 of gestation. Parallel data is not available in goat; however, this was in agreement with the Scotti *et al*. [9] in bitches. These investigators observed continuous increase in the value of peak systolic velocity in queen. Similarly, Di-salvo *et al.*, [8] observed same pattern of waveform in the bitch. Value of (EDV) increased from day 39 to 120 of gestational non-significantly, which did not find parallel literature, but was not in agreement with data in bitch reported by Scotti *et al*. [9]. In current study, PI increased from day 39 to 60 of gestation and then decreased up to 90 days of gestation. All these changes in PI were in agreement with Serin *et al*. [16]. When comparison of the present result was made with the normal canine and feline umbilical artery, similar PI patterns were found. In contrast to canine [8,21] and queen [9] pregnancy PI values reached the constant stage at about 90 days of gestation, and there were no more changes until parturition in goat. During the whole study, RI values were decreased non significantly. Results were different than those reported by Serin *et al*. [16]. It might be due to breed differences or due to the angle difference of transducer at the time of observation.

The umbilical vein blood flow pattern was almost flat with slightly wavy margin during all weeks of gestation without any abnormality with fetal outcome. These observations are in accordance with human medicine [18-20], and veterinary medicine [8,16].

## Conclusion

In conclusion, result of current study showed that changes in the blood flow parameters like PI, RI, PSV and EDV during gestation may be helpful in assessment of antenatal development of fetus and the fetal perfusion during pregnancy in goat. With further studies based on the current study may be helpful in the evaluation of any abnormality in goat pregnancy including intrauterine growth restriction of fetuses and other fetoplacental abnormalities during gestation.

## Authors’ Contributions

KK, RKC and RAL have designed the study and planned the research experiments. KK performed the research experiments. RKC supervised the research. SK, TCJ, SoK and SarK help in conducting experiment. All authors read and approved the final manuscript.
